# Associations of Job Insecurity With Perceived Work-Related Symptoms, Job Satisfaction, and Turnover Intentions: The Mediating Role of Leader–Member Exchange and the Moderating Role of Organizational Support

**DOI:** 10.3389/fpsyg.2020.01329

**Published:** 2020-07-07

**Authors:** Giovanni Di Stefano, Gaetano Venza, Davide Aiello

**Affiliations:** Department of Psychology, Educational Science and Human Movement, University of Palermo, Palermo, Italy

**Keywords:** job insecurity, leader–member exchange, organizational support, perceived health, work-related symptoms, job satisfaction, turnover intentions

## Abstract

This study wants to examine effects of job insecurity on several work-related outcomes (perceived work-related symptoms, job satisfaction, and turnover intentions) by developing a moderated mediation model. The model emphasizes the role played by the quality of leader–member exchange (LMX) in mediating the relation between perceived job insecurity and outcomes related to work, and the moderating role of perceived organizational support (POS) in influencing the mediation. Survey data from 510 workers at Italian organizations were collected, and regression was used to evaluate the hypotheses. After age, gender, education, and organizational tenure were controlled, results showed that perceived quality of LMX carried the effect of job insecurity on all outcomes, and that this relationship was stronger for employees who reported higher levels of POS. This study makes important theoretical and practical contributions to job insecurity, LMX, and POS research, underlining the importance of promoting the leader–member relationship’s quality in an ethical and supportive work environment.

## Introduction

An increasing body of research focusing on the associations between types of contract and negative psychological responses has emphasized the relation between job insecurity, health, and different work outcomes, such as well-being conditions (Benavides et al., 2000; Virtanen et al., 2002; Yaşlıoğlu et al., 2013; Griep et al., 2015). Job insecurity is a psychosocial risk associated with adverse impacts for both the worker and the organizational context. It has serious consequences for employees and is associated with the intent to leave the organization (Vander Elst et al., 2014; Probst et al., 2018). A meta-analysis on job insecurity outcomes showed that job insecurity has negative effects on several job and organizational attitudes and health (Sverke et al., 2002).

However, we cannot automatically infer that job insecurity directly relates to several work-related outcomes. For example, De Witte (2005), while emphasizing the effect that job insecurity has, i.e., on well-being, also underlines that among other psychological risks or job demands, job insecurity did not, however, represent the most troublesome factor. Also, Loi et al. (2011) showed that job insecurity did not seem to have a significant effect on performance, while Cheng and Chan (2008) found a significant negative association between insecurity and impaired performance. As such, research is needed to determine the conditions under which this occurs.

One specific concern about the effect of insecurity on work outcomes is the effect of job insecurity on supervisor–employee relationship. Researchers showed that leader–member exchange (LMX) may have an important role in worker well-being, job satisfaction, and turnover intentions (Griffeth et al., 2000; Lapierre and Hackett, 2007; Volmer et al., 2011), but what is needed is a deeper understanding of how social exchange elements and relationships (i.e., LMX) may intervene in the effect of job insecurity to work-related outcomes (Flickinger et al., 2016).

Further, studies have also shown that when the employee–organization relationship is undermined by distrust and lack of support, workers are more likely to feel unsatisfied about their occupation and consider leaving (e.g., Dulebohn et al., 2012).

**FIGURE 1:**
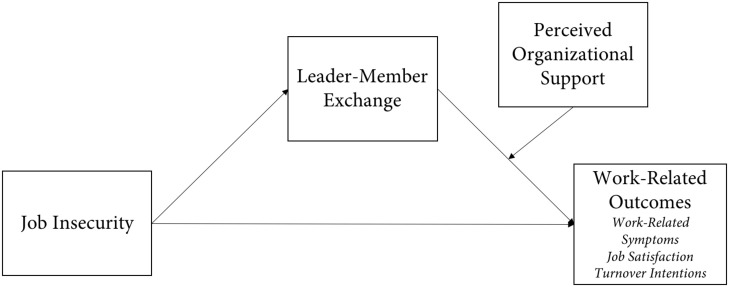
Proposed model for investigating the relationships among job insecurity, leader–member exchange, perceived organizational support, and perceived work-related symptoms, job satisfaction, and turnover intentions.

In line with this, the present contribution aims to underline the effect of job insecurity on several work-related outcomes (perceived work-related symptoms, job satisfaction, and turnover intentions), by developing a moderated mediation model. The model emphasizes the mediating role of the quality of LMX underpinning the association between job insecurity and work outcomes, and the moderating role of organizational support in influencing the mediation (see Figure 1).

Precisely, we suggest that LMX quality mediates the influence of insecurity on work-related outcomes. Second, we acknowledge that the strength of perceived organizational support (POS), as a contextual variable, has the potential to intensify the effect of LMX on work outcomes. We hypothesize that low levels of LMX quality following high levels of job insecurity will result in low job satisfaction, low perceived health, and high turnover intentions. Hence, we suggest that the influence of LMX on work outcomes will be greater for workers with higher POS levels, compared with those with lower POS.

## Theoretical Background and Hypotheses

### The Role of Leader–Member Exchange in Mediating the Relationship Between Job Insecurity and Work-Related Outcomes

Insecure workers are characterized by low levels of occupational health (Virtanen et al., 2002) and are subject to stronger exposure or show higher vulnerability to stress (Hall, 2006; Yaşlıoğlu et al., 2013), reduced organizational commitment (Sverke et al., 2002), and job satisfaction (De Witte and Näswall, 2003; Reisel et al., 2010). Job insecurity is assumed to have harmful consequences for workers as well as organizations, since it has a significant impact on workers’ stress and on the intention to leave (De Witte et al., 2010; Vander Elst et al., 2014; Probst et al., 2018).

An increasing amount of research has focused on the costs of job insecurity in the long term for well-being (Hellgren and Sverke, 2003; De Witte, 2005; Vander Elst et al., 2014). Researches indicate that job insecurity explains variations over time in both job satisfaction and physical pains. Extended periods of job insecurity decrease job satisfaction and well-being and increase physical symptomatology (Heaney et al., 1994; Hellgren and Sverke, 2003; Reisel et al., 2010).

Following De Cuyper and De Witte’s (2007) study, it may be argued that job insecurity can be considered as a violation of the psychological contract, which negatively affects job satisfaction and organizational commitment.

The LMX model (Graen and Uhl-Bien, 1995; Gerstner and Day, 1997) posits that each leader–follower dyadic pair develops relationships characterized by uniqueness, and that high-quality LMX relationships are beneficial to the follower in terms of mutual support, trust, and decision-making discretion.

Prior literature suggests that temporary employment discourages workers’ organizational commitment and loyalty (Zeytinoglu and Cooke, 2005; De Cuyper et al., 2008); also, followers’ perceptions of LMX positively influence job satisfaction, well-being, and health, and negatively intention to turnover (e.g., Dulebohn et al., 2012). Job insecurity may also threaten the exchanges other than the worker–organization relationship, so that it could be viewed as a discrepancy in the social exchange (Shoss, 2017). Additionally, LMX is more effective when workers perceive less security at work (Loi et al., 2011). Generally speaking, Dulebohn et al. (2012) showed that LMX is an important mediator involved in the relation between various antecedents and outcomes, appearing to bridge their association.

According to this, we hypothesize that:

H1: LMX quality mediates the relationship between job insecurity and (a) work-related symptoms, (b) job satisfaction, and (c) intention to turnover.

### Moderating Role of Organizational Support

As Joelson and Wahlquist (1987) pointed out, job insecurity has a harmful impact on well-being and job satisfaction because of unpredictability and uncontrollability. First, job insecurity is related to unexpectedness, since what will occur is uncertain, making it problematic to respond and act in a proper way. Also, unpredictability implies the inability to control the threats (De Witte, 1999).

Given that organizational support facilitates adaptation in transitions, by providing the employees with help against psychological consequences of job loss (i.e., Schlossberg and Leibowitz, 1980), one can expect that positive organizational support may help workers to cope with uncertainty, also promoting organizational commitment although under conditions of high job insecurity (Di Stefano et al., 2018; Venza and Cascio, 2019).

Perceived organizational support (Eisenberger et al., 1986) is the worker’s perception of how the organization cares about employees’ expectations and needs, appreciates their contributions, is willing to help, supporting, and rewarding. While LMX refers to social exchanges with supervisors, POS refers to social exchanges with the organization (e.g., Settoon et al., 1996; Wayne et al., 1997). Since workers who have access to high quality exchanges with their organization (i.e., perceive higher organizational support) should benefit to a greater extent from high LMX quality, one may argue that high LMX employees should be more satisfied, and even less prone to turnover intentions if they have high, as opposed to low, POS (Erdogan and Enders, 2007). POS should increase the strength of the LMX-job satisfaction relationship. So, we hypothesize the following:

H2: POS moderates the strength of the relationship between the LMX quality and (a) work-related symptoms, (b) job satisfaction, and (c) intention to turnover, such that the relationship is weaker when POS is high rather than low.
H3: POS moderates the strength of the indirect relationship of job insecurity with (a) work-related symptoms, (b) job satisfaction, and (c) intention to turnover via LMX, such that the mediated relationship is weaker when POS is low than under high.

## Materials and Methods

### Sample and Procedure

Participants were 510 employees (45.1% were females) from four mobile services call centers located in Italy. These organizations regularly survey employees about their well-being and perceived working conditions, while a committee discusses results to consider proposals for organizational development. Data for the present research came from one of these comprehensive surveys. All employees received e-mail providing detailed information regarding research, the requirements for inclusion, the link to survey, and the assurance of confidentiality. In order to reduce common method bias (Podsakoff et al., 2003), the survey did not request any personal information, and the order of item presentation was counterbalanced across the respondents.

Age ranged between 21 and 59 years, with an age of 31.88 years on average (*SD* = 11.57), and the average job tenure was 10.97 years (*SD* = 8.12). As for educational qualification, the largest part (72.8%) had a high school diploma.

### Measures

#### Job Insecurity

Perceived job insecurity was assessed by five items adopted from Job Content Questionnaire (Karasek et al., 1998). Sample items included “My job is secure.” Respondents specified their extent of agreement on a four-point Likert scale, ranging from 1 (“Strongly disagree”) to 4 (“Strongly agree”) and were re-coded so that more agreement corresponds to more job insecurity perception. The coefficient alpha was 0.84.

#### Leader–Member Exchange

Quality of working leader–follower relationships was assessed by the seven-item LMX-7 questionnaire (Graen and Uhl-Bien, 1995). Sample items included “How well does your leader recognize your potential?” Participants provided their responses using five-point scales, each of which is different from one item to another (1 = “Rarely,” 5 = “Very often,” or 1 = “None” to 5 = “Very high”). Higher scores represent a higher quality exchange between the supervisor and the employee. The coefficient alpha was 0.96.

#### Perceived Organizational Support

Organizational support was assessed by the short version (eight items) of the POS scale from Eisenberger et al. (1986, 1997), and Muse and Stamper (2007). Sample items included “The organization takes pride in my accomplishments at work.” Responses were recorded on a five-point agreement scale, ranging from 1 (“Strongly disagree”) to 5 (“Strongly agree”). Higher scores represent a higher perceived support from the organization. The coefficient alpha was 0.98.

#### Perceived Work-Related Symptoms

Perceived work-related symptoms were measured with Multidimensional Organizational Health Questionnaire’s list of eight psychosomatic symptoms (Avallone and Paplomatas, 2005). Respondents were asked to evaluate how frequently over the past 6 months they perceived several symptoms, using a four-point Likert scale ranging from 1 (“Never”) to 4 (“Often”), and then assessing what percentage they attribute these disorders to the work performed. Sample items included “In the last 6 months, how often have you perceived… Muscle and joint pains?” Higher scores represent a larger amount of perceived symptoms related to work. The coefficient alpha was 0.92.

#### Job Satisfaction

Job satisfaction was assessed using the three-item measure of global job satisfaction proposed by Cammann et al. (1983) and Bowling and Hammond (2008). An example item is “All in all I am satisfied with my job.” The measure uses a five-point Likert scale from 1 (“Strongly disagree”) to 5 (“Strongly agree”). Higher scores represent a higher job satisfaction. The coefficient alpha was 0.91.

#### Turnover Intentions

Intention to quit the job was measured with Mobley et al.’s (1978) three-item measure of intention to quit. An example item is “As soon as possible, I would leave this organization.” The scale uses a five-point Likert scale, ranging from 1 (“Very unlikely”) to 5 (“Certain”). High scores reflect high turnover intentions. The coefficient alpha was 0.92.

### Data Analysis

Before proceeding with the analyses of the hypothesized relations between variables, several confirmatory factor analyses (CFAs) were performed using the maximum likelihood estimation method in AMOS 20 (Arbuckle, 2011) to examine the distinctiveness of the latent variables and examine the existence of common method bias and alternative model specifications (Podsakoff et al., 2003). First, a measurement model was examined including six latent variables: job insecurity, LMX, POS, work-related symptoms, job satisfaction, and turnover intentions, using scale items as observed indicators explained by the latent factors and allowing the factors to correlate which each other. Next, the hypothesized model with six correlated factors was compared to other alternative models: a model with three correlated factors that integrates job insecurity, LMX, and POS items into one dimension, and specifies outcomes as separate constructs; a three-correlated factor model that specifies job insecurity, LMX, and POS as separates constructs, and integrates all item outcomes into one dimension; a single overall latent factor model, underlying all the items designed for the questionnaire. Due to the number of items measured for some constructs, partial disaggregation technique was used by combining items into composites in order to reduce higher levels of random error; hence, items that relate to job insecurity, LMX, POS, and perceived work-related symptoms were combined to create two composite indicators of each construct instead of several single-item indicators.

Hypotheses were tested with Model 4 and Model 14 of Hayes’ (2013) SPSS macro PROCESS for estimating moderated mediation effects, following the approach described in Preacher et al. (2007) and Hayes (2018). Model 4 was used to test the mediating role (H1) of LMX along with the direct relationship between job insecurity and work-related outcomes. Model 14 was used to simultaneously test whether the POS moderated the relationship between LMX and work-related outcomes (moderator hypothesis, H2), and if the indirect path was moderated by POS (moderated mediation hypothesis, H3). The macro PROCESS uses bootstrapping (*n* = 5000) to estimate unstandardized coefficients and biased corrected confidence intervals (CIs) to assess results in a single step. Age, gender, education, and organizational tenure were inserted as control variables.

## Results

### Measurement Models

Confirmatory factor analyses provided support for the hypothesized six-correlated-factor model; results indicate acceptable model fit to the data (see Table 1). This model provided better statistical significance compared with the alternative model in which all predictor items are loaded onto a single factor [Δχ^2^ (9) = 2142.467, *p* < 0.001]; the fit indexes were also better than the fit from the model in which work-related outcomes items are loaded onto a single factor [Δχ^2^ (9) = 714.918, *p* < 0.001]; and better than single overall latent factor model [Δχ^2^ (15) = 3433.063, *p* < 0.001]. Thus, the results of alternative CFA models provided evidence of construct independence.

**TABLE 1:** Fit statistics for measurement model comparison.

		χ^2^	df	CFI	TLI	RMSEA	[90% CI]	SRMR	χ^2^_diff_ (model comparison)
1	Six-factor model	112.256*	39	0.988	0.980	0.061	0.048–0.074	0.016	
2	Three-factor model (predictors)^a^	2254.723*	48	0.651	0.520	0.301	0.290–0.311	0.235	2142.467*(2vs.1)
3	Three-factor model (outcomes)^b^	827.174*	48	0.877	0.830	0.179	0.168–0.189	0.083	714.918*(3vs.1)
4	One-factor model	3545.319*	54	0.447	0.325	0.356	0.346–0.366	0.178	3433.063*(4vs.1)

*N* = 510. ^*a*^Job insecurity, leader–member exchange, and perceived organizational support as one factor. ^*b*^Perceived work-related symptoms, job satisfaction, and turnover intentions as one factor. *p < 0.001.

### Descriptive Statistics

The means, standard deviations, intercorrelations, and reliabilities for all the variables in this study can be seen in Table 2.

**TABLE 2:** Descriptive statistics and intercorrelations among the study variables.

		Mean	SD	1	2	3	4	5	6	7	8	9
**Control variables**												
1	Age	31.88	11.57	−								
2	Gender^a^	1.45	0.50	–0.04	−							
3	Education^b^	2.39	0.71	0.27*	0.02	−						
4	Tenure	10.97	8.12	0.49*	–0.01	0.30*						
**Predictor variable**												
5	Job insecurity	3.26	0.59	–0.04	0.03	–0.01	0.00	−				
**Mediator variable**												
6	Leader–member exchange	2.92	0.74	0.08	–0.03	0.04	0.00	−0.41*	−			
**Moderator variable**												
7	Organizational support	2.25	1.29	0.03	–0.05	–0.04	–0.02	−0.73*	0.39*	−		
**Outcome variables**												
8	Work-related symptoms	1.87	1.35	0.07	–0.01	0.12*	0.29*	0.41*	−0.52*	−0.45*	−	
9	Job satisfaction	2.78	1.20	−0.12*	–0.06	–0.04	−0.25*	−0.50*	0.57*	0.50*	−0.83*	–
10	Turnover intention	3.73	1.07	−0.12*	0.03	0.03	−0.16*	0.54*	−0.59*	−0.56*	0.62*	−0.62*

*N* = 510. ^*a*^1 = male, 2 = female; ^*b*^from 1 = “middle school graduation” to 5 = “postgraduate qualification.” *p < 0.01.

As shown in Table 2, the intercorrelations showed, first, that control variables were uncorrelated, in most cases, with the constructs; only education was weakly correlated with work-related symptoms (*r* = 0.12), and organizational tenure was weakly correlated with work-related symptoms (*r* = 0.29), job satisfaction (*r* = −0.25), and turnover intentions (*r* = −0.16). Second, job insecurity correlated with work-related outcomes: it showed a moderate positive correlation with work-related symptoms (*r* = 0.41) and turnover intentions (*r* = 0.54), and a moderate negative correlation with job satisfaction (*r* = −0.50); also, it was moderately and negatively correlated with LMX (*r* = −0.41) and strongly and negatively correlated with POS (*r* = −0.73). Mediator variable, i.e., LMX, correlated moderately and negatively with work-related symptoms (*r* = −0.52) and turnover intentions (*r* = −0.59), and moderately and positively with job satisfaction (*r* = 0.57), and with the moderator variable (i.e., organizational support: *r* = 0.39). Last, organizational support moderately correlated with all three outcomes, i.e., negatively with work-related symptoms (*r* = −0.45) and turnover intentions (*r* = −0.56), and positively with job satisfaction (*r* = 0.50).

### Test of Hypotheses

#### Mediation Hypothesis

Hypothesis 1 predicted that LMX mediated the relationship of job insecurity with work-related outcomes (work-related symptoms, job satisfaction, and turnover intentions). After controlling for covariates, the results showed that job insecurity had indirect effects on independent variables via LMX in the expected direction (*b* = 0.39 for work-related symptoms, *b* = −0.36 for job satisfaction, and *b* = 0.33 for turnover intentions), and in all cases, bootstrapped 95% CI did not include zero ([0.30, 0.48] for work-related symptoms; [−0.45, 0.28] for job satisfaction; [0.26, 0.41] for turnover intentions) (see the upper part of Table 3). Consequently, LMX partially mediated the job insecurity-outcomes relationship. Hence, Hypothesis 1 was supported.

**TABLE 3:** Mediation and moderated mediation analyses.

	Work-related symptoms						Job satisfaction				Turnover intentions				
				
				95% CI					95% CI					95% CI	
												
Predictors	*b*	SE	*p*	LL	UL	*b*	SE	*p*	LL	UL	*b*	SE	*p*	LL	UL
	**Results from the mediation model (Model 4)**														

Job insecurity^a^	0.54	0.09	<0.001	0.37	0.71	–0.64	0.07	<0.001	–0.78	–0.50	0.64	0.06	<0.001	0.52	0.76
LMX^b^	–0.78	0.07	<0.001	–0.91	–0.64	0.72	0.06	<0.001	0.60	0.83	–0.66	0.05	<0.001	–0.76	–0.56
Indirect effect^c^	0.39	0.05	<0.001	0.30	0.48	–0.36	0.04	<0.001	–0.45	–0.28	0.33	0.04	<0.001	0.26	0.41
*R*^2^	0.40*					0.31*					0.50*				
*F*	56.55					45.31					82.22				

	**Results from the moderated mediation model (Model 14)**														

LMX	–0.68	0.07	<0.001	–0.81	–0.54	0.66	0.06	<0.001	0.54	0.77	–0.61	0.05	<0.001	–0.71	–0.51
POS	–0.29	0.05	<0.001	–0.39	–0.19	0.20	0.04	<0.001	0.11	0.28	–0.22	0.04	<0.001	–0.29	–0.14
LMX x POS	0.36	0.06	<0.001	0.25	0.47	–0.18	0.05	<0.001	–0.27	–0.08	–0.01	0.04	0.75	–0.10	0.07
*R*^2^	0.47*					0.50*					0.53*				
*F*	55.79					62.87					69.79				

**N* = 510. LMX, leader–member exchange; POS, perceived organizational support. Covariates (age, gender, education, and tenure) are omitted for parsimony. *B* represents unstandardized regression coefficients with ordinary least squares (OLS) regression method. ^*a*^Effect of job insecurity on dependent variable controlling for LMX (path *c’*). ^*b*^Effect of LMX controlling for job insecurity (path *b*). ^*c*^Indirect effect of job insecurity on dependent variable (path *ab*). ^∗^*p* < 0.001.*

#### Moderation Hypothesis

**FIGURE 2:**
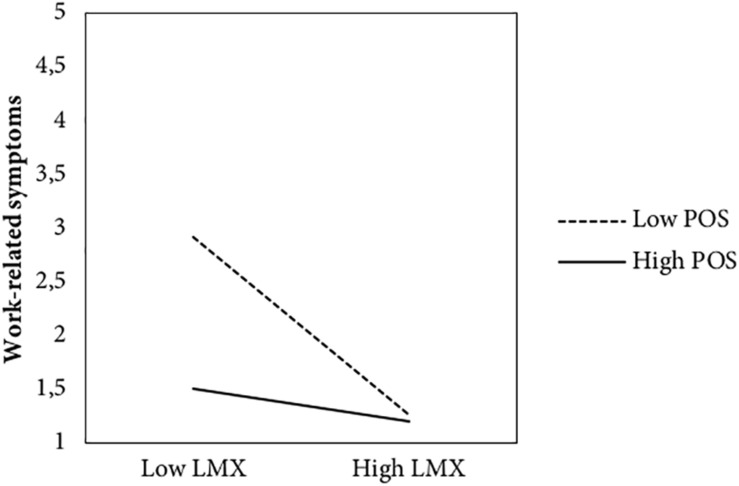
Impact of leader–member exchange on work-related symptoms at low and high levels of perceived organizational support. *Note:* LMX, leader–member exchange; POS, perceived organizational support.

**FIGURE 3:**
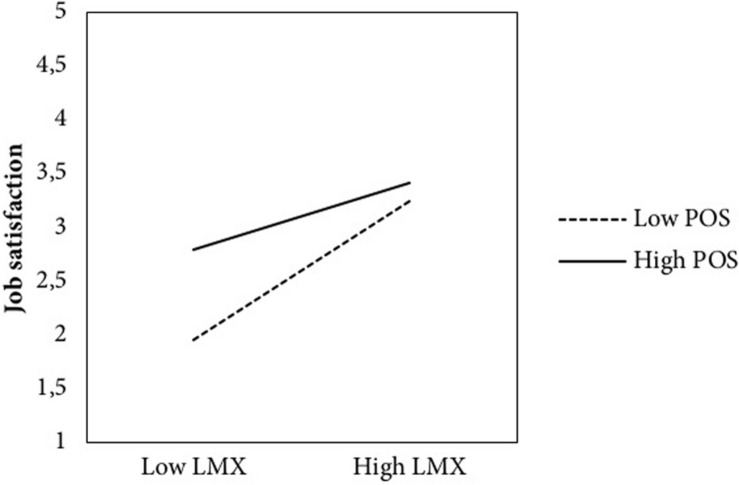
Impact of leader–member exchange on job satisfaction at low and high levels of perceived organizational support. *Note:* LMX, leader–member exchange; POS, perceived organizational support.

Hypothesis 2 predicted that POS moderated the relationships of LMX with work-related outcomes. As can be seen in the lower part of Table 3, the job insecurity × LMX interaction was found to be significant in predicting work-related symptoms (*b* = 0.36) and job satisfaction (*b* = −0.18) but was non-significant in predicting turnover intentions (*b* = −0.01). To probe the pattern of significant moderation effects, the relation between LMX and work-related symptoms, and LMX and job satisfaction were plotted across different values of the moderator (i.e., POS). Test of simple slope revealed that the negative LMX-symptoms relationship was stronger (*t* = -12.33, *p* < 0.001) under low POS (-1 SD), but weaker (*t* = −2.00, *p* = 0.04) under high POS (+1 SD) (see Figure 2). Similarly, but in the opposite direction (see Figure 3), simple slope test revealed that the positive LMX-job satisfaction relationship was weaker (*t* = 11.18, *p* < 0.001) under low POS (-1 SD), but stronger (*t* = 4.75, *p* < 0.001) under high POS. Thus, H2 was partially supported, highlighting the role of POS in moderating the effect of the relationship between LMX and two out of three work-related outcomes.

#### Moderated Mediation Hypothesis

According to Hypothesis 3, the indirect effects of job insecurity on work-related outcomes via LMX were moderated by POS. Results showed that the indirect effect of job insecurity on work-related symptoms through LMX was stronger for those with low POS (effect = 0.57, 95% CI [0.44, 0.70]), while it was weaker for those with high POS (effect = 0.11, 95% CI [0.04, 0.18]) (see Table 4). Moreover, the moderated mediation index was significant, yielding a value that did not include 0 in the CI (index = −0.18, 95% CI [−0.24, −0.13]). Also, results revealed that the job insecurity exerted an indirect effect on job satisfaction through LMX and that it was more remarkable for those with low POS (effect = −0.44, 95% CI [−0.55, −0.33]), while it was weaker for those with high POS (effect = −0.21, 95% CI [−0.30, −0.15]) (see Table 4). Furthermore, the moderated mediation index yielded a value that did not include 0 in the CI (index = 0.09, 95% CI [0.05, 0.13]). Finally, the conditional indirect effect of job insecurity on turnover intentions was non-significant (index = 0.01, 95% CI [0.02, −0.03]) (see Table 4). Consistent with H3a and H3b, these results confirmed that the mediated effect of the perceptions of job insecurity on work-related symptoms and job satisfaction via LMX was dependent on POS levels, while the conditional indirect effect of job insecurity on turnover intentions (H3c) was not confirmed.

**TABLE 4:** Moderated indirect effects.

			95% CI	
			
Conditional indirect effects (through LMX)	*Coefficient*	SE	LL	UL
**Work-related symptoms**				
POS (−1 SD)	0.57	0.07	0.44	0.70
POS (+1 SD)	0.11	0.04	0.04	0.18
	Index			
Index of moderated mediation	–0.18	0.03	–0.24	–0.13
**Job satisfaction**				
POS (−1 SD)	–0.44	0.06	–0.55	–0.33
POS (+1 SD)	–0.21	0.04	–0.30	–0.15
	Index			
Index of moderated mediation	0.09	0.02	0.05	0.13
**Turnover intentions**				
POS (−1 SD)	0.30	0.04	0.23	0.38
POS (+1 SD)	0.32	0.05	0.22	0.43
	Index			
Index of moderated mediation	0.01	0.02	–0.04	0.07

**N* = 510. LMX, leader–member exchange; POS, perceived organizational support.*

## Discussion

The purpose of this study was to examine the relationship between job insecurity and several work-related outcomes. As expected, we found that employees higher in job insecurity reported higher levels of perceptions of work-related symptoms and lower job satisfaction, and are more likely to express higher intention to turnover. This is line with the previous studies, which revealed that temporary workers show worse physical and psychological health conditions compared to those with permanent contracts (Benavides et al., 2000; Virtanen et al., 2002; Hall, 2006; De Witte et al., 2015).

The main objective of our research was to assess the mediating role of LMX in the association of job insecurity with work-related outcomes. Congruent with our hypotheses, we find that higher LMX had a mediating effect on lower levels of perceived work-related symptoms, higher job satisfaction, and lower intention to turnover.

Previous research has highlighted that LMX is associated with poor well-being and job satisfaction (e.g., Dulebohn et al., 2012). Nevertheless, limited research has been conducted concerning the conditions under which low LMX quality have their worst effects. This study attempts to fill this gap by estimating whether the strength of the associations between LMX and work-related outcomes is conditional upon worker POS. Results from regression analyses revealed that POS moderates the relationship of LMX with work-related symptoms and job satisfaction. Particularly, workers who feel low support from their organization are more susceptible to perceive more symptoms related to their work activity and being unsatisfied of their job as a result of low levels of LMX quality. This finding is consistent with Loi et al.’s (2011) results that insecure workers were more responsive to the support gained from supervisors.

Finally, we found that POS moderates the indirect effect of job insecurity through LMX on two out of three work-related outcomes, namely, the work-related symptoms and the job satisfaction. These findings highlight that low LMX quality, following from high job insecurity, is more likely to escalate into work-related symptoms and into lowering job satisfaction under low POS levels; conversely, detrimental and mediated effects of job insecurity on well-being and job satisfaction via LMX may be further attenuated under high POS condition.

Therefore, LMX and organizational support should be considered when developing organizational intervention programs and strategies intended to promote employees’ health perceptions, in particular under higher job insecurity condition, since the importance of considering the subjective dimension of this construct (e.g., De Witte, 2005) improves the quality of exchanges in organizations and thus increases favorable work-related factors, protecting them from negative ones.

It is worth mentioning some limitations to this study, mainly due to its cross-sectional nature, which makes it difficult to infer causal relations among variables, albeit the causal relations were derived from theoretical constructs and previous research. Therefore, further research should consider prospective longitudinal studies over time. However, recently Spector (2019) has effectively argued how “longitudinal design to reflect causality has been overstated and that it offers limited advantages over the cross-sectional design in most cases in which it is used” (Spector, 2019, p. 125). Also, it is worth nothing that some intercorrelations between constructs range from moderate to high. Nevertheless, CFAs provided support for the hypothesized six-correlated-factor model. So, despite the high correlations between some variables, the constructs are not identical and it is possible to consider them separately.

Anyway, these results have several implications for HR management. Particularly in times of economic crisis, when permanent contracts are difficult to obtain, results showed that stimulating the LMX, under the more general condition of a perceived support from the organization, could increase satisfaction and health. From this point of view, the quality of LMX becomes more important when job security is inadequate; under this condition, high-quality LMXs become crucial for organizations attempting to improve employees’ well-being and satisfaction. In line with our results, when workers have feelings of job insecurity, the promotion of LMX under high levels of POS is particularly effective.

## Data Availability Statement

The datasets generated for this study are available on request to the corresponding author.

## Ethics Statement

Ethical review and approval was not required for the study on human participants in accordance with the local legislation and institutional requirements. The patients/participants provided their written informed consent to participate in this study.

## Author Contributions

GD contributed to conceptualization, writing—original draft preparation, and data analysis. GV contributed to writing—review and editing. DA contributed to data collection and writing—review. All authors contributed to the article and approved the submitted version.

## Conflict of Interest

The authors declare that the research was conducted in the absence of any commercial or financial relationships that could be construed as a potential conflict of interest.
